# The New COVID-19 Related Psychological Distress Pandemic

**DOI:** 10.3390/jcm11010237

**Published:** 2022-01-02

**Authors:** Michele Roccella, Gioacchino Lavanco, Luigi Vetri

**Affiliations:** 1 Department of Psychology, Educational Science and Human Movement, University of Palermo, 90128 Palermo, Italy; michele.roccella@unipa.it (M.R.); comunicazioni@gioacchinolavanco.it (G.L.); 2 Oasi Research Institute-IRCCS, Via Conte Ruggero 73, 94018 Troina, Italy

Although a few years have passed since the beginning of the COVID-19 pandemic, a large body of scientific literature is already present on the impact that the worldwide spread of the virus has had on people’s quality of life.

The studies conducted on previous decade’s epidemics, such as SARS, MERS and the H1N1 flu, as well as the research conducted the day after other types of catastrophes, have shown us the possible psychopathological implications linked to unexpected and massive events, which threaten the health and safety of individuals and undermine the stability of communities.

A total of 50 studies, 48 research articles and 2 reviews, included in the Special Issue “The Impact of the COVID-19 Emergency on the Quality of Life of the General population”, fully reflect the complexity and the psychological and health implications caused by the unprecedented health, economic and social emergency provoked by the pandemic spread of the SARS-CoV-2 virus.

The interesting research included in the Special Issue essentially concern three macro-areas.

The first area of interest includes the analysis of the neuropsychological implications determined by the COVID-19 pandemic. These studies already clearly showed that the effects of the rapid spread of an epidemic go well beyond the morbidity and mortality of the infected people, strongly affecting various areas of the entire population’s daily life, with inevitable repercussions on mental health [1,2,3,4,5].

The second macro-area analyzed by the studies of this Special Issue concerns the health measures necessary to face the coronavirus emergency, in particular in relation to prevention, treatment, and subsequent follow-up measures. In this phase of coexistence with COVID-19, the territory and its resources represent the crucial place for controlling the epidemic but, at the same time, also the place where new opportunities can be found for prevention and health promotion interventions [6,7,8,9,10].

The third macro-area of interest regarded the effects of the COVID-19 pandemic on the management and evolution of other diseases. Unfortunately, fear is becoming a reality. In an attempt to limit the damage caused by COVID-19, the right to health and care of people with other serious diseases is further jeopardized [11,12,13,14,15].

The outbreak of COVID-19 evidenced the vulnerability of the health system and of the patients with chronic or rare diseases; it also highlighted their needs in terms of access to care, adhesion to a therapy schedule, and home care.

The COVID-19 pandemic represents a catastrophe of global dimensions, the first traumatic event on a global scale in the most recent history of humanity. Although the degree of individual exposure to a traumatic event such as this is extremely variable because of different environmental, personal and professional factors, it can be considered in some ways an “in vivo experiment” in the psychopathology of the masses; heterogeneous for geographical, economic and cultural reasons, the world population is exposed in an extreme and persistent way to the same trauma in a short time.

In summary, the articles in this Special Issue reiterate how the unprecedented health, economic and social emergency that our society is experiencing is causing an increase in mental distress in subjects with a pre-existing psychiatric pathology, particularly in health workers, but also in the general population. In particular, the prevalence of symptoms of anxiety, depression, and post-traumatic stress disorder is clearly increasing in the general population, and the scientific community is called to give answers in order to avoid the current viral pandemic turning into a psychological distress pandemic.
